# Health economics modeling of antiretroviral interventions amongst HIV serodiscordant couples

**DOI:** 10.1038/s41598-021-93443-x

**Published:** 2021-07-07

**Authors:** Haisheng Wu, Qiuyan Yu, Liping Ma, Lin Zhang, Yuliang Chen, Pi Guo, Peng Xu

**Affiliations:** 1 Department of Preventive Medicine, Shantou University Medical College, No. 22 Xinling Road, Shantou, 515041 China; 2 Department of Preventive Medicine, School of Public Health and Management, Wenzhou Medical University, University Town, Wenzhou, 325035 China; 3 Hengrui Pharmaceutical Co., Ltd., No. 7 Kunlun Mountain Road, Lianyungang Economic and Technological Development Zone, Lianyungang, Jiangsu China; 4 Zhoukou Center for Disease Control and Prevention, No.10 Taihao Road East Section, Zhoukou, Henan China; 5 National Center for STD/AIDS Prevention and Control, Chinese Center for Disease Control and Prevention, No. 155 Changbai Road, Beijing, 102206 China

**Keywords:** HIV infections, Health care, Health occupations

## Abstract

Antiretroviral treatment (ART) and pre-exposure prophylaxis (PrEP) for HIV-serodiscordant couples, effectively reduce mortality, transmission events and influence quality of life at the expense of increased costs. We aimed to evaluate health economics of antiretroviral-based strategies for HIV-serodiscordant couples in the China context. A deterministic model of HIV evolution and transmission within a cohort of serodiscordant couples was parameterized using the real-world database of Zhoukou city and published literature. We evaluated the mid-ART (a historical strategy, initiating ART with CD4 < 500 cells/mm^3^), early-ART (the current strategy, offering ART regardless of CD4 cell counts) and a hypothetical strategy (early-ART combined short-term daily PrEP) versus the late-ART (the baseline strategy, initiating ART with CD4 < 350 cells/mm^3^) offered by 2008 national guidelines. We estimated the incremental cost-effectiveness ratios (ICER) and incremental cost-utility ratios (ICUR) from a societal perspective, derived by clinical benefits and HIV-caused life quality respectively, and portrayed their changes over a 0–30 year’s timeframe. The model projections indicated that the antiretroviral-based interventions were more likely to obtain clinical benefits but difficult to improve quality of life, and cumulative ICER and ICUR were generally decreasing without achieving cost-saving. Scale-up access to ART for the HIV-positive among serodiscordant couples was easily fallen within the range of paying for incremental life-years and quality adjusted life years by the societal willingness. The hypothetical strategy had the potential to prevent most seroconversion events within marriages but required enormous upfront costs, thus it took a long time to reach established thresholds. The current strategy of early-ART is the most cost-effective. Clarifying the obstacles of high cost of PrEP and improving life quality for HIV-serodiscordant couples have emerged as an urgent requisition.

## Introduction

HIV-serodiscordant couples, one partner is seropositive and the other is seronegative, are the substantial-risk group susceptible to transmission and the priority population for HIV treatment and prevention^1,2^. On the one hand, the latest guidelines of the World Health Organization (WHO) in 2016 recommend offering antiretroviral treatment (ART) for all HIV-seropositive partners regardless of CD4 cell count and oral pre-exposure prophylaxis (PrEP) for HIV-seronegative partners at high risk of HIV seroconversion^3^. China has been committed to expanding the scope of ART since it launched the national free antiretroviral treatment program (NFATP) in 2003. From 2012, all the seropositive in HIV-serodiscordant couples had been eligible to voluntarily receive free ART regardless of their CD4 cell counts^4^. On the other hand, the promotion and application of PrEP in China have been slow. As such, The Chinese government is planning to take pivotal steps forward. For example, the Chinese Center for Disease Control and Prevention (CDC) is uniting China Medical University to conduct studies on large-scale implementation of PrEP, dedicating to developing guidelines for the implementation of PrEP in China. In such a context some studies prospectively applied mathematical models to estimate the cost-effectiveness of PrEP in men who have sex with men in China^5,6^.

Substantial evidence has demonstrated the clinical and preventive benefits of antiretroviral-based interventions including early ART and PrEP. ART scale-up for HIV-serodiscordant couples simultaneously benefits in HIV suppression for seropositive partners and prevents onward transmissions in their seronegative partners, has been demonstrated in both clinical trials and real-world settings^7–15^. PrEP is a time-limited instead of life-long intervention, unlike ART, adapted as a “bridge” to curb seroconversion events before suppression of viral replication in the HIV-positive partners during periods where they have not yet initiated ART or may not achieve sustained viral suppression^2^. PrEP is also available daily or on demand^6,16^, so time-limited and intermittent PrEP is more appropriate in resource-limited configurations.

Early ART and PrEP are still the key public health approaches to combat the epidemic of HIV in the future^1^. As we know that there is a global prospect proposed by the United Nations to achieve the 90–90–90 target of HIV/AIDs by 2020 and transform it from pandemic to sporadic endemic by 2030^3^. Nonetheless, the implementation of ART or PrEP is not satisfactory due to the obstacles to the implementation of these antiretroviral-based interventions strategies. For example, most HIV-positive patients need to take a long period from the diagnosis to ART initiation^17^. The obstacles might responsible for that most countries will not meet these commitments at the appointed time^18^.

Crucially, for antiretroviral-oriented strategies, the relationship between investment costs and the clinical benefits as well as the quality of life for HIV patients has yet to be examined simultaneously in real-world setting. A recent study has appointed out that HIV patients are declining the life quality^19^. Several models confirmed that early initiation of ART, time-limited PrEP, and their combination may be cost-effective for high-risk groups^20–23^. In China, there is an evidence gap in health economic assessment for the antiretroviral-based strategies. Due to the wide assumptions of model input parameters and target populations across countries^24^, these findings cannot allow being extrapolated to China, where has low HIV prevalence nationwide, high HIV endemic, different economic level and culture background.

**Figure 1:**
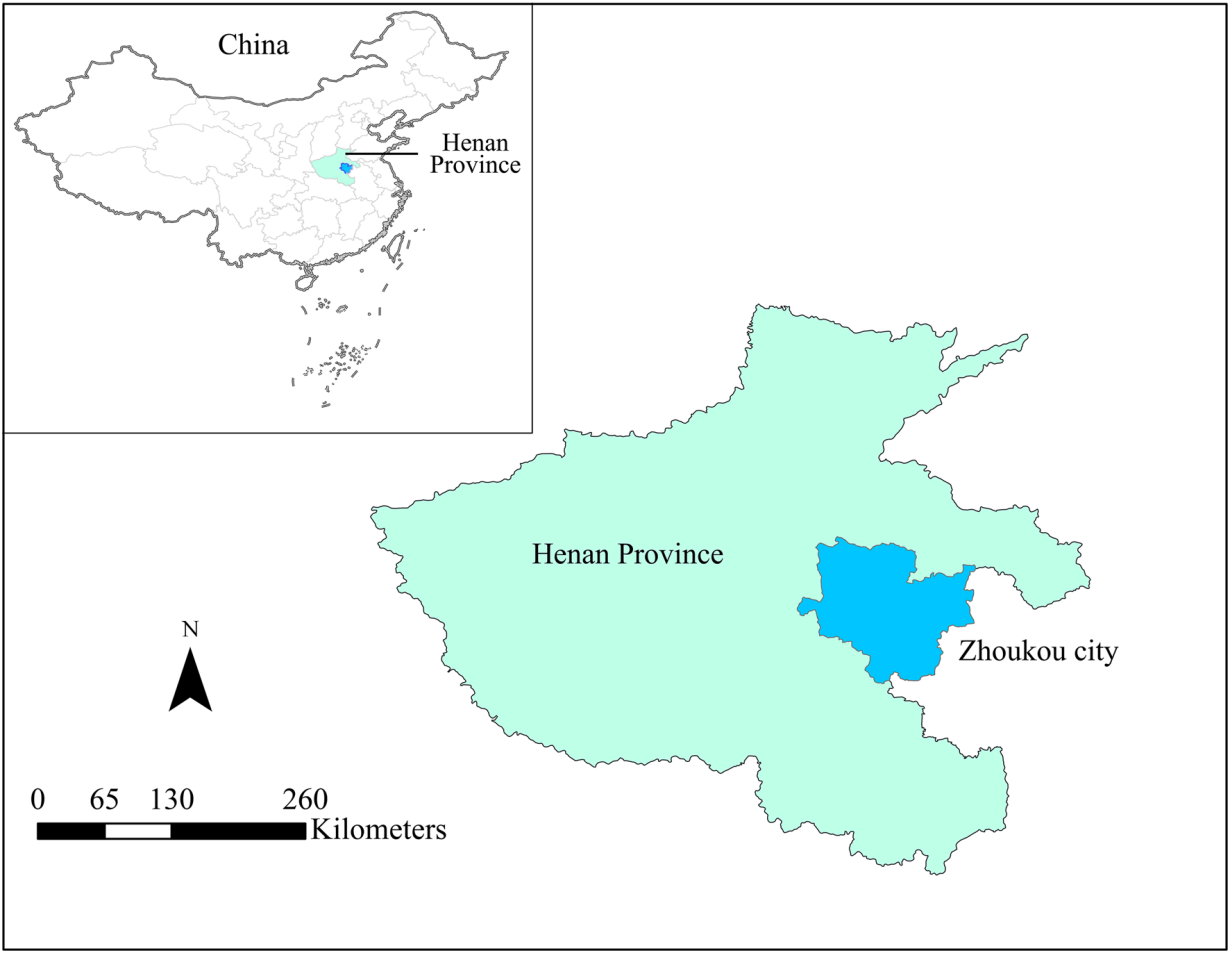
Geographical location of Zhoukou city of Henan province in China. We used the ArcGIS version 10.2 (ESRI, Redlands, CA, USA; https://www.esri.com/) to create the map.

Here, we utilized the real-world database of Zhoukou city to simultaneously assess cost-benefits from the perspective of clinical benefits and life quality for HIV-serodiscordant couples. Zhoukou city (Fig. 1), as one of the six vital epicenters of the HIV epidemic in Henan province, is a typical area of HIV epidemic in Henan province and has similar HIV epidemic characteristics to Henan Province. Henan is a rural province in China that bears a high HIV/AIDS burden, where had the top HIV/AIDs incidence rates in 2004 but the lowest relative increase in the past 10 years attributed to the strong support of the nation and NFATP program^25^. Nonetheless, Henan was the only province with HIV/ADSs incidence exceeded 1.0 per 100,000 from 2004 to 2014^25^. Henan is also one of China's less developed and agriculture-oriented provinces, resulting in a challenge of medical infrastructure. Furthermore, there are no consistent estimates of the seroconversion rates amongst HIV-serodiscordant couples in Henan province. A systematic review and meta-analysis in China showed that the overall HIV seroconversion rate in Henan during 2005–2011 was 0.9 per 100 person-years (PY)^26^. But a regional study in Henan revealed that the overall seroconversion rate during 2006–2012 was 0.59/PY, in which the seroconversion rate was 0.43/PY amongst couples that positive partners have initiated ART and 5.87/PY whereas positive partners were untreated^11^.

In this study, a Markov decision model consisting of disease module and transmission module was adapted and extended to reproduce HIV evolution and transmission. To fill the evidence gap, we conducted the health economics evaluation of the historical and current strategies of scale-up access to ART and a hypothetical strategy of 1-year PrEP for HIV-serodiscordant couples with a variable timeframe in Zhoukou city, China.

## Materials and methods

### Subject involvement

Information of HIV-serodiscordant couples was extracted from the database of Zhoukou HIV/AIDs monitoring system and medical system. With informed consent, 1251 HIV-serodiscordant couples were given a questionnaire survey by professional staff of the local centers for disease control and prevention in 2015. Data used in this work were anonymous, and no individual identifiable information was available in our study. Patients or the public were not involved in the design, or conduct, or reporting, or dissemination plans of our research. There are no plans of disseminating research results to participants. All methods were performed following the Declaration of Helsinki.

### Study settings and data resource

#### CD4-based states

According to treatment status and CD4 cell counts of the HIV-seropositive, we divided the disease progression status into three period (9 health states in total), including the disease status before ART initiation (pre-ART period), the disease status during ART (on-ART period) and the absorption status (death). All health states were defined based on the following CD4 cell count: > 500 cells/mm^3^ in pre-ART (S1); 350–499 cells/mm^3^ in pre-ART (S2); 200–349 cells/mm^3^ in pre-ART (S3); < 200 cells/mm^3^ in pre-ART (S4); > 500 cells/mm^3^ on ART (S1′); 350–499 cells/mm^3^ on ART (S2′); 200–349 cells/mm^3^ on ART (S3′); < 200 cells/mm^3^ on ART (S4′); and Death (D).

#### Intervention strategies

**Figure 2:**
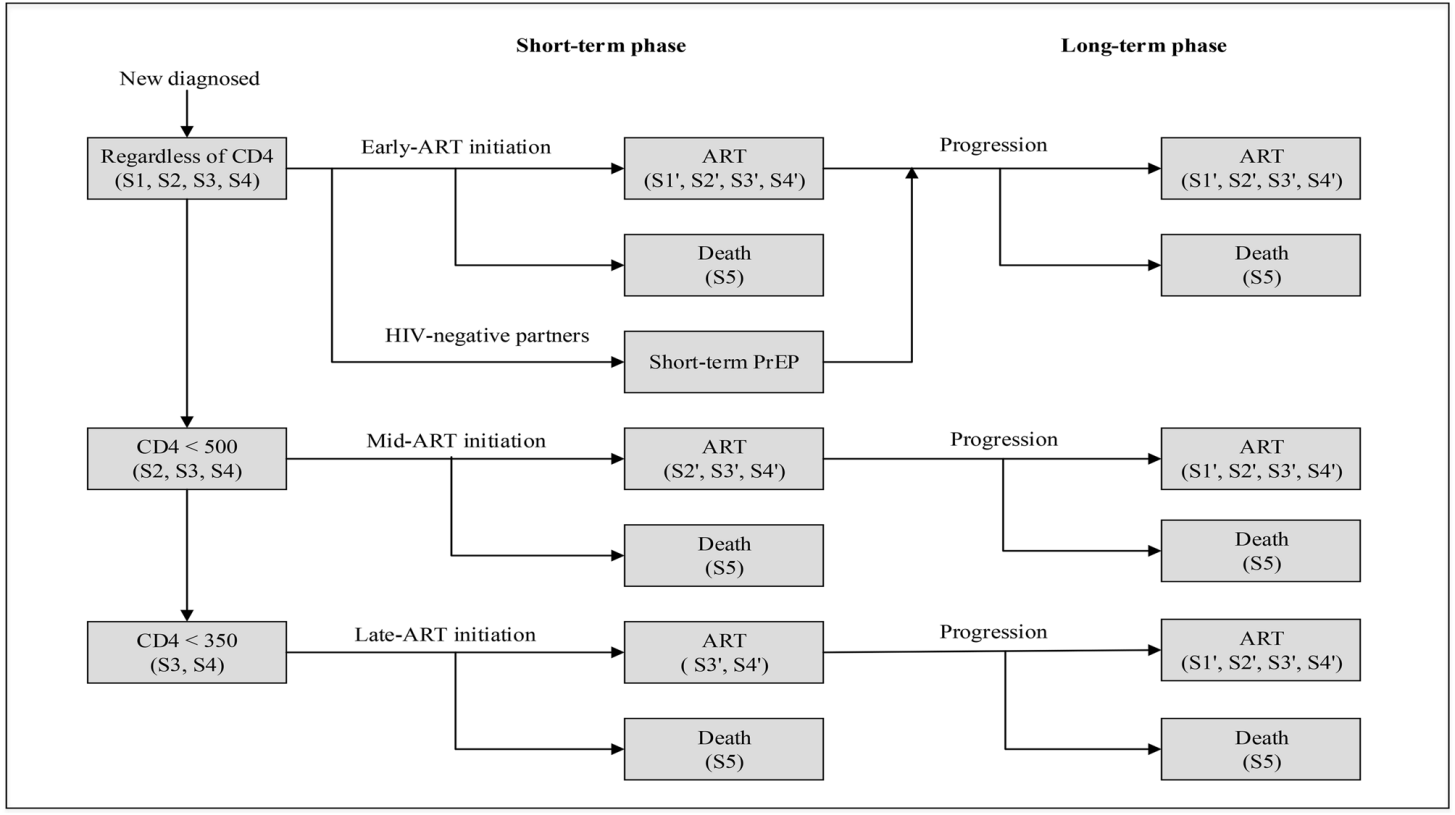
The flow chart of intervention strategies. We divided the disease progression status into three periods (9 health states in total). Symbols refer to health stages of HIV infection: S1 = in pre-ART period with CD4 count of more than 500 cells/mm^3^; S2 = in pre-ART period with CD4 count of 350–500 cells/mm^3^; S3 = in pre-ART period with CD4 count of 200–349 cells/mm^3^; S4 = in pre-ART period with CD4 count of less than 200 cells/mm^3^. S1′ = in on-ART period with CD4 count of more than 500 cells/mm^3^; S2′ = in on-ART period with CD4 count of 350–500 cells/mm^3^; S3′ = in on-ART period with CD4 count of 200–349 cells/mm^3^; S4′ = in on-ART period with CD4 count of less than 200 cells/mm^3^; S5 = death. The model assumed diagnosis upon progression to 30 years after this diagnosis. For each HIV-serodiscordant couple, the positive partner would experience diagnosis period, pre-ART period and on-ART period in sequence in the model simulation, and the negative partner in the cohort of short-term PrEP combined early-ART would be offered 1-year PrEP from the diagnosis of their partners, while the negative partner in other cohorts of late-ART, mid-ART and early-ART would receive no intervention. Upon meeting the criteria for ART initiation of different intervention strategies, the HIV-positive would start ART. HIV-negative partners would also receive specific ART initiation strategies after the occurrence of seroconversion events.

We aimed to conduct a health economics evaluation of historical and current strategies of scale-up access to ART and a hypothetical strategy of PrEP. Based on the evolution and progress of WHO recommendations and national guidelines, the antiretroviral-based strategies considered in this study were: late-ART (initiating ART at CD4 < 350 cells/mm^3^); mid-ART (initiating ART at CD4 < 500 cells/mm^3^); early-ART (initiating ART to all HIV-seropositive partners regardless of CD4 cell count). We additionally considered a hypothetical strategy: short-term PrEP + early-ART (providing early-ART to all HIV-seropositive partners and offering daily PrEP drugs to seronegative partners to curb transmission events prior to sustained viral suppression in their seropositive partners). The late-ART was set as the reference in the case of multiple intervention strategies. A schematic of the targeted intervention strategies is shown in Fig. 2. Among all strategies, the study samples were in pairs, containing the HIV-positive and their marital but HIV-seronegative partners. Those HIV-positive partners would be partitioned by sex and initial health states based on demographic characteristics from real-world databases, with their partners were all regarded as seronegative at the start of model simulation. Then the HIV-positive would experience diagnosis period, pre-ART period and on-ART period in sequence in the model simulation. The HIV-negative in short-term PrEP + early ART cohort would be offered 1-year PrEP from the diagnosis of their partners while HIV-negative partners in other cohosts received no intervention. Upon meeting the criteria of ART initiation of different intervention strategies, the HIV-positive would accept ART treatment. HIV-negative partners would also receive specific ART initiation strategies after the occurrence of seroconversion events.

#### Data collection

The real-world database of HIV-serodiscordant couples of Zhoukou city from 2003 to 2015 was used in our study. Zhoukou city is one of the six vital HIV/AIDS prevention and treatment areas in Henan province with a relatively completed database of HIV-serodiscordant families. There are 2785 HIV-serodiscordant couples in this database.

The professional staff from the local centers for disease control and prevention conducted a survey for 1251 HIV-serodiscordant couples from the database in 2015. The survey samples included the HIV-positive who received treatment and those who did not, distributing in different health states. The WHO Quality of Life Questionnaire for Brief Version (WHOQOL-BREF) was used to evaluate HIV-related life quality^27,28^ of the HIV positive partners and a field questionnaire survey was applied to obtain the information that included partial direct non-medical cost paid for individuals and government. The direct medical cost, calculated by invoking the relevant outpatient and hospitalization records in the medical system, mainly included ART cost and additional medical expenses due to opportunistic infections.

### Analytic overview

We applied a Markov decision model of HIV disease, treatment and transmission^29,30^ to compare early-ART, mid-ART and short-term PrEP + early-ART with late-ART for HIV-serodiscordant couples. The model predicted the following outcomes within a variable timeframe from a societal perspective: clinical outcomes (the survival rates and mean life expectancy), HIV-related quality of life measured by quality-adjusted life year (QALYs), transmission outcomes (seroconversion events amongst serodiscordant couples), and economic outcome (per-person total cost). We assessed intervention strategies from the perspective of clinical benefits measured by per-person life expectancy and quality of life benefits measured by per-person QALYs. Base on per-person life expectancy, we calculated the adjusted incremental cost-effectiveness ratio (ICER) of early access to ART and PrEP, as the difference in total cost divided by the difference in life expectancy, accounting for the health benefits, transmissions and costs for the HIV-serodiscordant couples (see the Supplementary Material file). Base on QALYs, we estimated the adjusted incremental cost-utility ratio (ICUR) in the same way (see the Supplementary Material file).

Based on the WHO principle of cost–benefit^31^, we classified intervention strategies as very cost-effective with the ICER/ICUR lower than the per capita GDP of Zhoukou in 2015 ($3797)^32^, as cost-effective with the ICER/ICUR lower than three times the GDP ($11,391). A strategy that simultaneously reduced overall costs and increased life years or QALYs was considered cost-saving. We discounted costs, life years and QALYs for each pair at an annual rate of 3%, but did not discount the number of survival and transmissions^33,34^.

### Markov decision model

#### Disease module

The disease module as a part of the Markov decision model is a computer-based, state-transition model of HIV treatment and progression in resource-limited settings, with the ability to simultaneously track HIV-related clinical outcomes and the resources linked with these outcomes^30^. The HIV-positive progressed based on key parameters drawn from the real-world database to reflect the natural history of HIV. Disease progression was estimated by a mathematical function of underlying CD4 count divided into the nine health states and transition probabilities between states, and differentiated between the HIV-positive at pre-ART and on-ART period.

The well-established methods to plan a reasonable health resource allocation need to precisely estimate the rate of disease progression or the transition probabilities among health states. We applied a multivariable multi-state Markov (MMSM) model, a mathematical model that combines multiple regression model and multiple states Markov model^35^, to estimate yearly state-transitions of the disease progression of both pre-ART and on-ART period. The eligible couples would be included in the MMSM model from the database if they were (1) HIV-serodiscordant couples (one partner was HIV-seropositive and the other was HIV-seronegative) at the baseline visit, (2) meeting the legal marriage age in China (male are over 22 years old, female are over 20 years old), (3) in a stable marriage without divorce or separation, (4) willing to provide informed consent, and (5) with at least one additional recorded for follow-up HIV test results for both partners. There were 2096 pairs of couples that meet the criteria were extracted from the database and their basic characteristics were presented in Table S1 in the Supplementary Material file. Specifically, the MMSM model firstly emphasized changes in yearly transition probabilities by calendar year and age (Figure S1 and Figure S2) and allowed for an explanation about factors on CD4 improvement and deterioration at treatment period (Table S2). Then two matrixes containing adjusted transition probabilities of pre-ART and on-ART period respectively were estimated, correcting the effect of factors that had a significant impact on state transitions (Tables S3 and S4). These transition probabilities represented precise transition probabilities between disease states we need in the disease module.

Additionally, when HIV carriers met strategy-specific treatment criteria, ART was initiated, with their health states would transform instantaneously and accordingly (e.g. a patient at S1 would change to S1′ once he or she was offered ART). The details of the disease module are provided in the Supplementary Material.

#### Transmission module

We used a transmission module, as a function of sex, time-dependent transmission rates and age-dependent duration of risk, to capture yearly seroconversion events of HIV-negative partners^33^. To do this, it applied deterministically the average cost trajectory, survival trajectory and trajectory of life quality determined by the Markov disease module to newly infected partners.

The overall transmission rates of HIV-serodiscordant spouses were not consistent among different intervention strategies^9^. By initiating ART therapy as early as possible to achieve sustained viral suppression or offering PrEP, the HIV-seronegative partners are exposed to high-risk situations for less time. Previous studies have revealed the gender differences in HIV transmission among heterosexuals^36^ and a national retrospective study in China of heterosexual partners indicated that females were more likely than males to transmit the virus to their seronegative partners, irrespective of treatment status^9^. HIV transmission between couples refers to the seropositive partners who transmit HIV during sexual intercourse, which is age-dependent and thus we set the duration of transmission based on the average age of the study population. Furthermore, the transmission module was allowed to assign the seroconvert partners to corresponding ART strategies, whose initial CD4-based states would be randomly assigned according to distribution characteristics. The details of the transmission module are provided in the Supplementary Material.

### Model parameter input

#### Study cohort

Table 1 shows the characteristics of the base-case cohort, reflecting the descriptive demographic of all HIV-serodiscordant couples from the HIV real-world database in Zhoukou city. We firstly set the mean age to 43 and sex proportion (male 54.91% and female 45.09%). Based on the CD4 count of HIV-positive patients at the time of the initial diagnosis, we classified them into the initial states (states S1–S4) mentioned above, followed which we estimated the distribution of initial health states. At the beginning of the model simulation, all positive individuals were at pre-ART period, and all negative partners are in a status of complete health without the acquisition of HIV. The further details of HIV progression parameters for different antiretroviral-oriented strategies are shown in Table S5–S7.

**Table 1:** Base-case input parameters of the disease model.

Variable	Base-case value for male	Base-case value for female	Range
**Cohort characteristic**			
Age (year)	43	43	
Sex (%)	54.91	45.09	
**Initial distribution of health states (%)**			
S1	11	10	
S2	14	14	
S3	30	31	
S4	45	45	
Other states	0	0	
Rate of relationship dissolution	0	0	0–0.5
**Transmission**			
Duration of transmission (year)	5	5	
Duration of PrEP drug (year)	1	1	
PrEP efficacy (%)	90	90	0.86–0.96
**Costs**			
**Yearly cost of different states ($)**			
S1	774.81	774.81	Base case × 0.5–2.0
S2	1009.63	1009.63	Base case × 0.5–2.0
S3	1178.31	1178.31	Base case × 0.5–2.0
S4	800.79	800.79	Base case × 0.5–2.0
S1′	3148.93	3148.93	Base case × 0.5–2.0
S2′	3439.13	3439.13	Base case × 0.5–2.0
S3′	3878.52	3878.52	Base case × 0.5–2.0
S4′	3426.45	3426.45	Base case × 0.5–2.0
Death	0	0	
General unemployment rate (%)	4.1	4.1	
Unemployment rate of the HIV-positive (%)	20.5	20.5	16.4–24.6
Annual rate of income growth (%)	8.5	8.5	6.5–12.1
Duration of work (year)	15	15	
Yearly cost of daily PrEP drugs ($)	4290	4290	Base case × 0.8–1.2
**Life quality**			
Utility of different states			
S1	0.74	0.74	0.72–0.77
S2	0.74	0.74	0.72–0.77
S3	0.73	0.73	0.69–0.77
S4	0.72	0.72	0.67–0.78
S1′	0.71	0.71	0.67–0.72
S2′	0.69	0.69	0.67–0.71
S3′	0.66	0.66	0.65–0.68
S4′	0.65	0.65	0.63–0.68
Death	0	0	
Utility of the HIV-negative	0.85	0.85	0.75–1.00

#### Transmission

We firstly focused on two patterns of transmission going in opposite directions (HIV-positive husbands transmit to negative wives, and HIV-positive wives transmit to negative husbands). Early-ART and its combination with short-term PrEP by initiating ART for HIV-positive partners as early as possible, transmission rates between couples remain at a relatively low level after the virus is completely suppressed, which usually takes 6 months from ART initiation^2,23^. We set it to 1 year based on a conservative assumption, during which providing PrEP to negative partners reduces transmission events by 90%^12,22^.

The model assumed that sexual activity was age-dependent and the duration of transmission risk depended on the initial average age of the population^33,37^. Based on the mean age of HIV-positive people in the real database as 43, the average duration of the transmission risk was set to 5 years^33^. The model also took into account changes in the time scale of the transmission risk of intervention strategies. The real-world database of Zhoukou and parameters offered by a published nationwide cohort study^9^ were utilized to estimate sex- and time-dependent transmission rates mentioned above, more details can be referred to the Supplementary Material file and the estimated results are shown in Table S8.

#### Costs

We considered a total cost from a societal perspective, summing up the HIV-caused costs from government-payer, individual-payer and income loss due to unemployment. Specifically, we referred to the HIV-related cost paid for both individuals and governments as direct costs, obtained from a field survey of HIV-serodiscordant couples in Zhoukou city conducted by staff from the local center for disease control and prevention in 2015 and outpatient and hospitalization records offered by the health administration. The Chinese government administers ART programs for HIV serodiscordant couples and paid for direct prevention and treatment (e.g., CD4 and VL testing, ART drugs, etc.), and individuals are responsible for the cost of nonprevention and nontreatment (e.g., transportation, working hours loss, etc.). The specific classification of direct costs and the details of the calculation were presented in Table S9.

The estimates of these time-dependent costs per year, stratified by CD4-based states and treatment status (pre-ART or on-ART) were presented in Table 1. Additionally, the yearly cost of daily PrEP drugs was attained from a recent study in China^5^. By transmission module, we also considered loss averted due to intervention-attributable reducing of seroconversion events amongst HIV-serodiscordant couples.

We utilized employment data to calculated income loss averted due to antiretroviral-attributable productivity gains among HIV-serodiscordant families. We assumed that the average duration of continuous work is 15 years based on the average age. Furthermore, the general unemployment rate was 4.1%, the unemployment rate of the HIV-positive was 20.5% and its range was 4–6 times the general unemployment rate, and the income growth rate was 8.5%^32^.

#### Life quality

We applied QALYs that sum the product of the utility assigned to each health state and the average duration that an individual remains in that state, as an indicator for assessing the HIV-related life quality of HIV-serodiscordant families. The utility and corresponding 95% confidence interval as its range, stratified by treatment status and health states, was estimated by WHOQOL-BREF for 1251 HIV-positive individuals in and out ART (Table 1). The higher the CD4 cell count, the lower the chance of opportunistic infection or HIV-related disease, thus a high CD4 state has a higher utility value (e.g. the utility value of S1 is 0.74 versus the utility value of S4 is 0.72). However, once an HIV-positive individual enters treatment status from treatment-naïve status, the utility will decline automatically (e.g. the utility of S1′ is 0.71 versus the utility value of S1 is 0.74). A reasonable explanation is that the tedious treatment process consumes extra time and cost, coupled with social factors and psychological factors of patients resulted in a decline in life quality. We set the health utility for the death state to be zero, and the utility for the negative partner to be 0.85 instead of 1, because their partners’ HIV infection unavoidably had a negative impact on their quality of life.

### Sensitivity and scenario analyses

Compared with the base-case scenario implemented firstly and populated as shown in Table 1 as well as Table S8 in the Supplementary Material file, we added alternative scenarios to probe the uncertainty of key parameters and model structural. First, to establish the incremental impact of scale-up ART as well as 1-year PrEP, we reduced transmission rates between HIV-serodiscordant spouses by 50% as a low-risk scenario and increased transmission rates by 100% as a high-risk scenario. Second, our initial assumption that hypothesized the utility of seronegative partners is 0.85 was verified by a low utility scenario setting utility to 0.75 and a high utility scenario setting utility to 1. Third, to determine the influence of rates of relationship dissolution, we explored a series of scenarios regarding the relationship dissolution rate as 10%, 20%, 30%, 40% and 50% respectively. We held the other input parameters constant at base-case levels in the above alternative scenarios and observed the changes of ICER and ICUR in a variable time framework.

We also implemented a one-way sensitivity analysis to explore the impact of the cost, utility values and transmission-related variables on both overall ICER and ICER over a 30-year time horizon. The range of variation of these variables is shown in Table 1.

### Ethics approval and consent to participate

Ethical approval was obtained from the ethics committee of the National Center for STD/AIDS Prevention and Control, Chinese Center for Disease Control and Prevention. The approval number is X150317363.

## Results

### Clinical benefits

Early access to ART improved survival rate in HIV-positive partners (Table 2): compared with late-ART, mid-ART increased the 10-year rate of survival among index patients from 83.6 to 86.5% and the 30-year of survival from 64.9 to 67.6%; Early-ART, with more significant clinical benefits, increased the 10-year rate of survival among index patients from 83.6 to 87.6% and the 30-year of survival from 64.9 to 68.4%. The earlier the initiation of ART, the more HIV-related deaths among HIV-positive partners could be averted, accordingly aggrandizing the life expectancy. For both the early-ART strategy and the short-term PrEP + early-ART strategy, they were equally effective in improving clinical outcomes for the HIV-positive partners because they both provided early-ART to HIV-positive partners.

**Table 2:** Life expectancy, HIV transmission, costs, cost-effectiveness and cost-utility over 10-year horizon and 30-year horizon respectively. Life expectancy for the 10-year and 30-year period indicates the average duration of survival through 10 years and 30 years, respectively. QALYs gained for the 10-year and 30-year period indicate the average accumulative QALYs through 10 years and 30 years, respectively. The overall ICER (overall incremental cost-effectiveness ratio) was calculated as the difference in costs for the index patient plus the difference in the increased costs for HIV-negative partners due to transmission and PrEP drugs divided by the difference in life expectancy for the index patients plus the difference in life-years lost due to transmission, all discounted at 3% per year. The overall ICUR (overall incremental cost-utility ratio) was calculated as the difference in costs for the index patient plus the difference in the increased costs for HIV-negative partners due to transmission and PrEP drugs divided by the difference in QALYs for the index patients plus the difference in QALYs lost due to transmission, all discounted at 3% per year.

Intervention	HIV index patients					HIV-negative partners			Overall ICER	Overall ICUR
	Survival (%)	Life expectancy (year)	QALYs gained	Total costs per index patients (2015 US$)	Transmissions Per Index patients (n, %change)	Life-years lost due to transmission (year)	QALYs lost due to transmission	Total cost increase due to transmission (2015 US$)		
**10-year period**										
Late-ART	83.6	9.10	4.93	30,841.0	0.054	0.041	0.170	1474.1		
Mid-ART	86.5	9.28	5.00	32,302.9	0.044 (18.5%)	0.028	0.138	1302.2	6832.4^§^	12,870.2
Early-ART	87.6	9.35	5.03	32,800.7	0.036 (33.3%)	0.021	0.115	1112.5	5876.1^§^	10,003.01^§^
Short-term PrEP + early-ART	87.6	9.35	5.03	32,800.7	0.014 (74.1%)	0.006	0.037	4635.5	17,869.4	21,559.4
**30-year period**										
Late-ART	64.9	24.29	10.13	65,574.2	0.054	0.292	0.796	3365.9		
Mid-ART	67.6	24.99	10.40	67,666.8	0.044 (18.5%)	0.211	0.642	2903.2	2081.9^&^	3842.5^§^
Early-ART	68.4	25.23	10.50	68,302.8	0.036 (33.3%)	0.166	0.529	2436.4	1689.5^&^	2822.1^&^
Short-term PrEP + early-ART	68.4	25.23	10.50	68,302.8	0.014 (74.1%)	0.059	0.193	5149.0	3848.3^&^	4638.0^§^

^§^This indicates the values were lower than the cost-effective threshold.

^&^This indicates the values were lower than the very cost-effective threshold.

### Quality of life

Both early-ART and mid-ART have improved the life quality of HIV patients, as treatment coverage has been expanded to varying levels, compared to late-ART. Over 10-year period and 30-year period, the overall life quality of the index partners was highest in the early-ART group, with corresponding QALYs of 5.03 and 10.50 respectively. The QALYs gained of the mid-ART group and the early-ART group increased over time because it is compensated by the benefits of an increase of life expectancy due to the early access of ART, as compared with late-ART.

### Costs

Although early initiation of ART closed many opportunistic diseases, thus preventing a certain amount of expense of care, a savings that less than outweighed expenditure of expanding access to treatment. Consequently, for the HIV-positive partners, early-ART and short-term PrEP + early-ART had the highest per-person costs ($32,800.7 over the first 10 years and $68,302.8 over 30 years), followed by the mid-ART group and the late-ART group (Table 2). For seronegative partners, the cost for the short-term PrEP + early-ART strategy is the highest due to the availability of the PrEP drugs to all HIV-negative partners in the first year.

### Transmissions

After the 5-year risk period, compared with late-ART, short-term PrEP + early-ART significantly prevented most of the projected transmissions (74.1%) versus late-ART. Early-ART and mid-ART also reduced transmissions among HIV-serodiscordant couples versus late-ART by 33.3% and 18.5%, respectively (Table 2).

### Cost-effectiveness

With the model included income loss, transmission-related life years lost and costs accrued to correct for ICER, we showed the ICER of all concerned strategies over a 10-year horizon and a 30-year horizon respectively in Table 2. Early-ART was cost-effective ($5876.1) as well as mid-ART ($6832.4), while short short-term PrEP + early-ART was not cost-benefits ($17,869.4), over a 10-year period. And all invention strategies could be regarded as very cost-effective versus the late-ART over a 30-year horizon.

### Cost-utility

Analogously, income loss, transmission-related QALYs lost and costs accrued were used to rectify ICUR calculated in the model. In terms of quality of life, only the early-ART could be considered as cost-effective ($10,003.01) over a 10-year period; and over a 30-year time horizon, only the early-ART could be considered as very cost-effective ($2822.1), both mid-ART and short-term PrEP + early-ART were just cost-effective.

**Figure 3:**
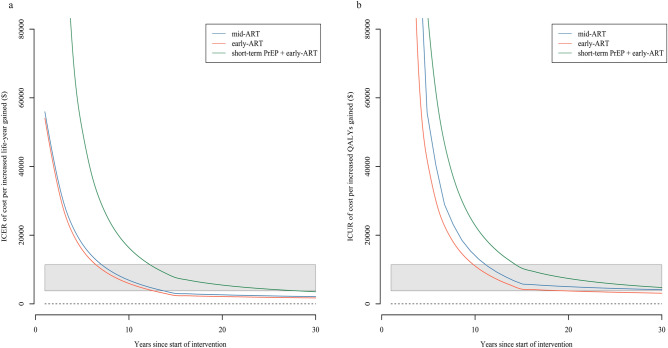
Outcomes of the intervention strategies at different time horizons. We calculated the incremental cost-effectiveness ratio (ICER) and incremental cost-utility ratio (ICUR) of targeted strategies compared with baseline strategies at 0–30 year’s horizon. (**a**) ICER of cost per life-year gained as a result of the model, for the three targeted strategies versus late-ART within HIV-serodiscordant couples. (**b**) ICUR of cost per QALY gained as a result of the model, for the three explored scenarios versus late-ART within HIV-serodiscordant couples. The grey band represents the $3797–11,391 threshold of cost-effectiveness of interventions and the black dotted line represents the cost-saving threshold (= 0).

We plotted the changes of ICER and ICUR overtime at a 30-year horizon (Fig. 3). In general, the ICER and ICUR of the early-ART and mid-ART were very close at different times, but both ICER and ICUR of early-ART were always lower, suggesting that the early access of ART is more cost-effective. The short-term PrEP + early-ART group had a significantly higher ICER and ICUR than other intervention strategies, because of the high cost of the PrEP drugs offered to HIV-negative partners. The ICER and ICUR of all strategies declined rapidly in the early period of the simulation, slowed down by the cessation of transmission within HIV-serodiscordant couples after 5 years, and leveled off after 15 years when income loss was no longer considered. Compared with ICER, the time of ICUR entering the cost-effective threshold was delayed, revealing that both of expanding access to ART and short-term PrEP were more likely to yield clinical benefits while improving quality of life for HIV-discordant families is more costly.

### Sensitivity analysis

**Figure 4:**
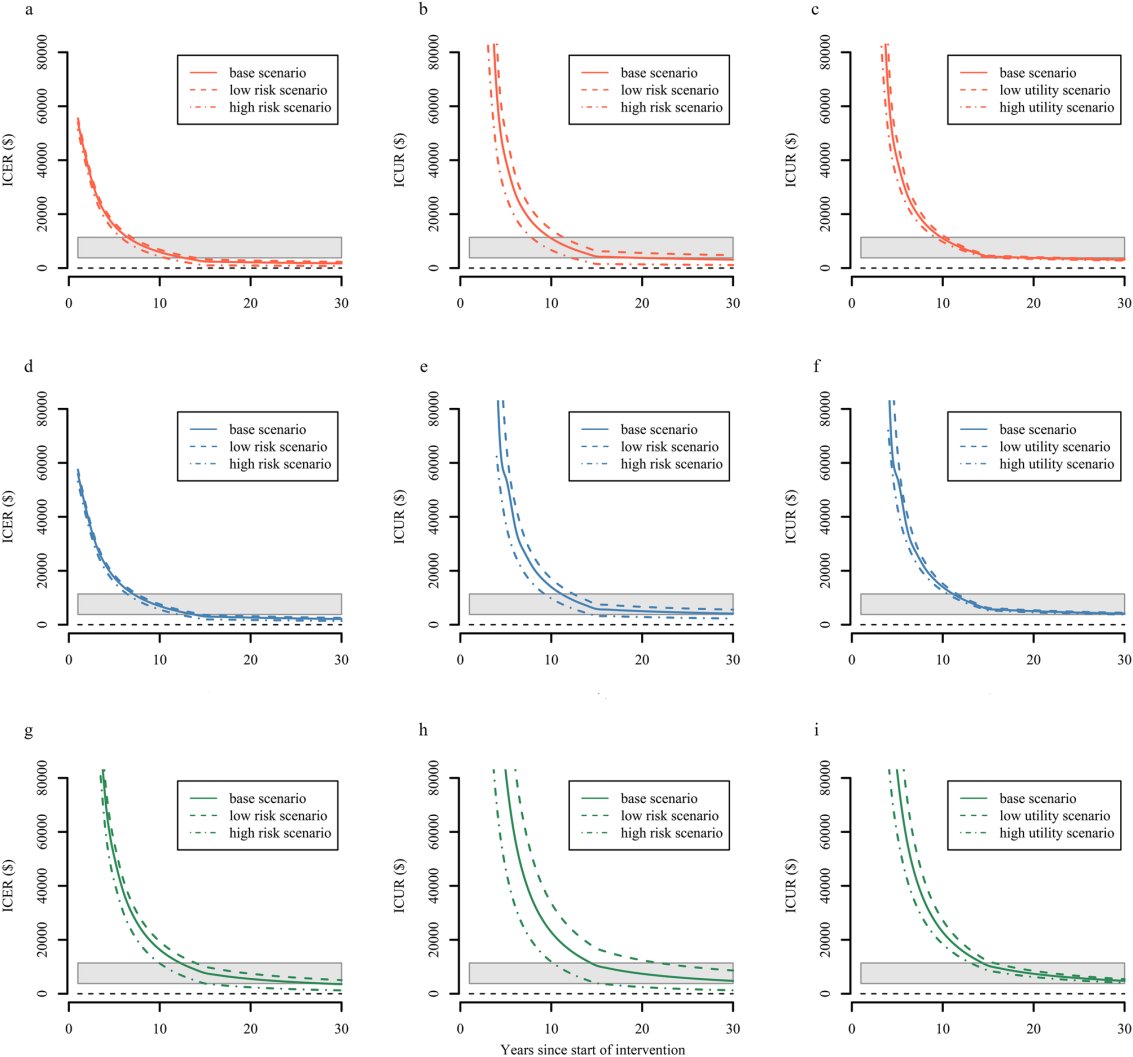
Outcomes of scenario analysis for transmission rates and the utility of seronegative partners. We explored the sensitivity of model results in high risk scenario (increasing the transmission rates to twice the base case), low risk scenario (reducing the transmission rates to half the base case), high utility scenario (increasing the utility of HIV-seronegative partners to 1) and low utility scenario (reducing the utility of HIV-seronegative partners to 0.75), respectively. (**a**) ICER varied from different levels of transmission risks among serodiscordant couples of early-ART; (**b**) ICUR varied from different levels of transmission risks among serodiscordant couples of early-ART. (**c**) ICUR varied from different levels of utility values of seronegative partners of early-ART. (**d**) ICER varied from different levels of transmission risks among serodiscordant couples of mid-ART. (**e**) ICUR varied from different levels of transmission risks among serodiscordant couples of mid-ART. (**f**) ICUR varied from different levels of utility values of seronegative partners of mid-ART. (**g**) ICER varied from different levels of transmission risks among serodiscordant couples of short-term PrEP + early-ART; (**h**) ICUR varied from different levels of transmission risks among serodiscordant couples of short-term PrEP + early-ART. (**i**) ICUR varied from different levels of utility values of seronegative partners of short-term PrEP + early-ART. The threshold for cost-effectiveness of $3797–11,391 is shown in the grey band, and the cost-saving threshold was shown by the black dashed line.

**Figure 5:**
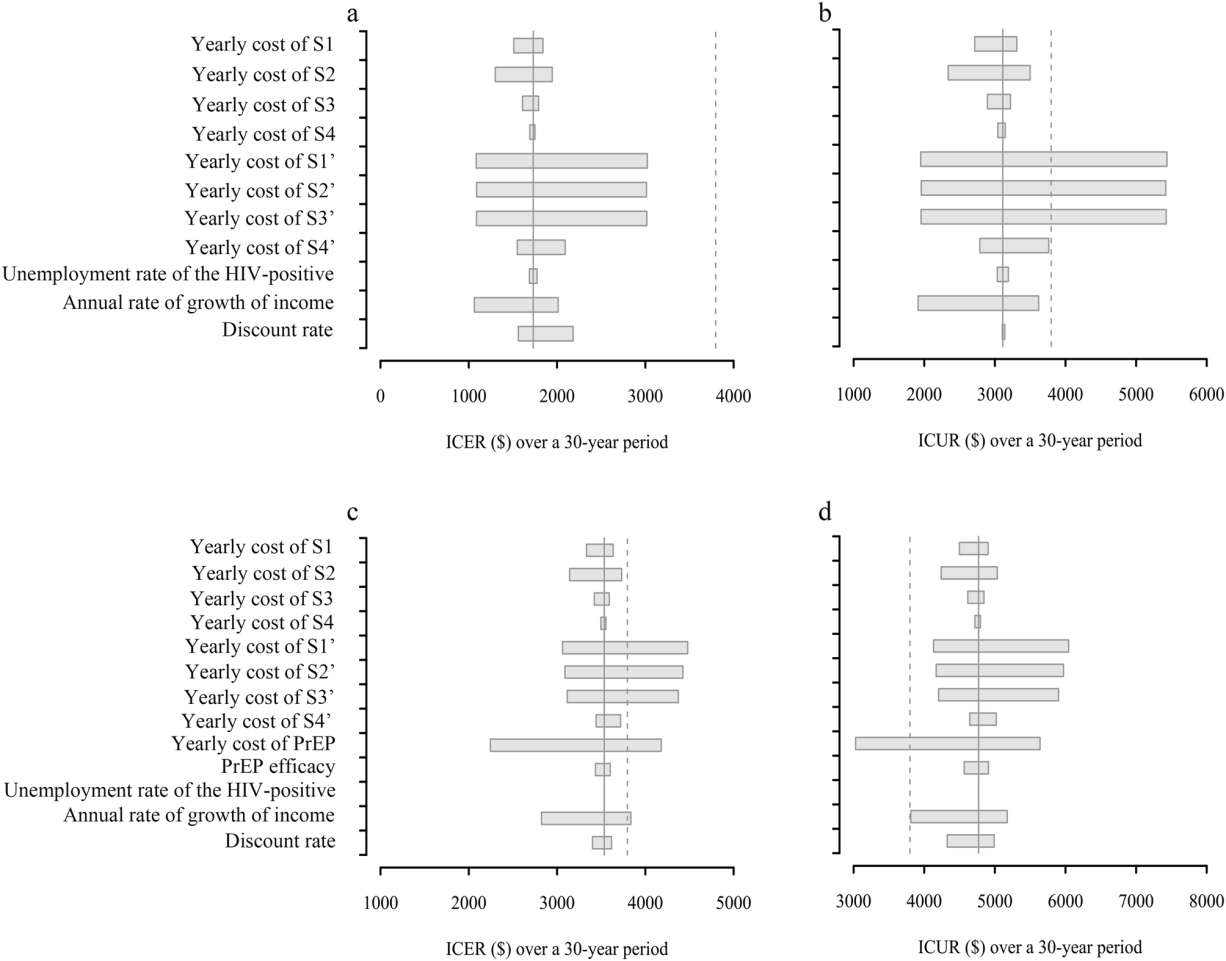
Outcomes of one-way analysis. We calculated the impacts of specific cost and transmission-related variables on (**a**) ICER in early-ART versus late-ART; (**b**) ICUR in early-ART versus late-ART; (**c**) ICER in short-term PrEP + early-ART versus late-ART; (**d**) ICUR in short-term PrEP + early-ART versus late-ART, over a 30-year period. The solid vertical grey line represents the results of the base case. The dashed vertical grey line represents the threshold ($3797) for being very cost-effective.

Over a 30-year horizon, projected ICER and ICUR of all targeted strategies were more cost-effective for the high-risk scenario in which there was a more reduction of seroconversion events, as compared with late-ART (Fig. 4). The low utility scenario and high utility scenario that changes the utility of seronegative partners only had a change on ICUR, but the corresponding impact is minimal, irrespective of intervention (Fig. 4). Another scenario analysis shows that reasonable changes of rates of relationship dissolution have little impact on study interpretation (Figure S3). Subtle differences in accumulative results caused by changes of variables over 30 years provided compelling evidence that the economic and policy conclusions based on our model were robust (Fig. 5, Figure S4 and Figure S5). It is worth noting that the cost of PrEP drugs had pronounced effects on projection derived by the model, suggesting that we can implement the short-term PrEP for HIV-serodiscordant partners more feasible and cost-effective by lowering the price of PrEP drugs. We also explored the relationship between the percentage reduction in the cost of PrEP and corresponding cost-effectiveness. The results indicate that the ICER of early-ART plus PrEP over 10-year period would reach the cost-effective threshold if there is a reduction in 39.0% of the cost of PrEP drugs; the ICUR over a 30-year period would become very cost-effective when the cost of PrEP reduces by 23.4%, while the ICUR over a 10-year period would only become cost-effective when the cost of PrEP reduces by 62.5% (Figure S6).

## Discussion

In China, there is sufficient evidence to support clinical and preventive benefits of antiretroviral-based interventions for HIV-discordant spouses^9,11^, but there is little research focusing on health economic problems regarding antiretroviral-based strategies and the relationship between cost and improvement in HIV-caused life quality. To fill this gap, we populated a mathematical model based on the real-world database of Zhoukou city and published studies to project the clinical benefits, life-quality, and economic effects of different intervention strategies for HIV-discordant spouses. Our model considered the historical and current strategies of early access of ART and a hypothetical strategy of short-term PrEP, which provided a reference for relevant decision-making departments.

The magnitude of generalizability of our results to couples elsewhere in China is noteworthy. Zhoukou is a remote and rural city with resource-limited settings. The HIV population of Zhoukou city is largely made up of middle-aged persons (i.e., the mean age is 43 at diagnosis) infected from unsterile conditions in plasma donation in the early years and have low reported rates of drug use or sexual promiscuity, similar to that in the whole Henan province^11^. The public health applicability of our results needs to consider the uniqueness of this population and our limited ability to verify some measures. The findings of this study may apply to the whole of Henan province and other rural regions with similar characteristics, such as Yunnan and Guangxi province^9^. However, the population uniqueness in this study limits the generalizability of our findings to groups with more different or multiple sources of HIV exposure, such as sex workers who use drugs and the men who have sex with men. Meanwhile, our results are also of limited applicability to those regions where is economically developed or HIV populations are dominated by drug abuse and sexual promiscuity. A previous study suggested that the consistency of effect ART intervention for serodiscordant couples in Henan province with elsewhere in China is more likely attributable to the government healthcare than the fact that they are from the same country^11^. This indicates the importance of taking economic and consumption levels into account in interpreting specific health economic assessments.

Expanding access to treatment has been an ongoing effort in the health care system and early-ART has been implemented as the current strategy. Our study proves that early-ART as the current strategy for HIV-serodiscordant couples is the most cost-effective. The characteristics of HIV-serodiscordant couples lead to the difficulty of a large-scale detection in China, so information about early HIV detection was not included in the database. In addition to the characteristics we mentioned in the introduction, the other reasons for the difficulty of large-scale HIV screening include the low availability of testing reagents due to low prevalence of HIV, the self-shame of HIV-positive individuals, and social discrimination^15^. Some studies have used mathematical models to analyze the cost-effectiveness of early HIV screening and combined HIV testing and treatment, and proved that these strategies are cost-effective in different research contexts^38–41^. Exploring the economic questions on frequent detection programs in China such as ‘test and treat’ and ‘universal testing’ amongst high-risk populations is the direction of future studies.

Short-term PrEP is considered as a 'bridge' to reduce the number of transmission events before viral suppression in the HIV-positive partners^12,23^ and the future choice in China^42^. But only two studies have discussed the cost-effectiveness of PrEP in MSM populations^5,6^, which draw a similar conclusion to our analysis that PrEP is difficult to be cost-effective due to the current high price. A combined intervention strategy of current ART and short-term PrEP for serodiscordant couples was considered and predicted based on our model, which provided scientific evidence for relevant decision-making departments. Short-term PrEP implementation is likely to be cost-effective if PrEP is given an intermittent basis or making generic PrEP drugs available to reduce total cost^14^. Our model predicted the cost-effectiveness of daily PrEP instead of other drug regimens such as on-demand PrEP, because there is a current lack of available data and parameters of other regimens.

This study has some limitations. First, based on a relatively conservative assumption that various dyads are independent, we limited the analysis to transmissions within serodiscordant marriages, but not including extramarital HIV infection and transmissions due to practical considerations as well as lack of parameters. Second, although drug resistance was not explicitly considered in our model, published studies have shown that the risk of acquired resistance is very low^43^. Third, as HIV-serodiscordant couples in the database of Zhoukou city have been identified, we conducted an analysis of HIV treatment and prevention based on the actual situation, instead of HIV screening or ‘test and treat’. Fourth, several important factors, such as viral load, condom use and sexual behavior that play a meaningful role HIV transmission, may be comparatively ignored in our model. For viral load, substantial evidence demonstrated that viral load is highly related to CD4, as both of them were strongly time-dependent during HIV evolution. Hence, considering only the CD4- or viral load-based transmission rate may help to simplify the model and improve efficiency for complicated mathematical models, as in previous studies^6,16^. Given that CD4 follow-up information in our data was more complete than viral load, we chose to build the model primarily with CD4. We were unable to account for condom use and sexual behavior in the model as they were not sufficiently available for the entire study population. Nevertheless, the model parameters (e.g., transition probabilities) derived from the real-world database actually included population-level information about the viral load, condom use and sexual behavior patterns, making the simulation results more consistent with the real world. Fifth, using annual updates might be too a long time frame to capture rapid changes in transmission rates following acute infection or treatment initiation. We endeavored to mitigate the impact of changes in viral load-related infectivity by estimating time-dependent transmission rates. Finally, this complex model is not easy to perform probabilistic sensitivity analysis^32^, although it has many benefits for further analysis of the model results.

In summary, our analysis suggested that the current strategy (i.e., early ART) is the most cost-effective in the Zhoukou context and the theme of the next step of the health systems is to improve life quality instead of mere survival for HIV-serodiscordant families. Considering that short-term PrEP has the potential to prevent most seroconversion events in HIV-serodiscordant spouses, the government could clarify the obstacles to offering PrEP with lower cost for large-scale implementation of short-term PrEP. Furthermore, the findings of this study may be applicable to the whole of Henan province and other similar remote regions in China. Further efforts should be directed to assess the public health approach reoriented to tackling existing challenges and to validate these results in resource-limited settings.

## Supplementary Information

Supplementary Information.

## Data Availability

Due to the need to protect personal privacy, data used in this work were anonymous, and all data are available on request. Please contact with Peng Xu (E-mail: xupeng2007@163.com).
