# Supplementary material for: Health economics modeling of antiretroviral interventions amongst HIV serodiscordant couples
Source: Sci Rep. 2021 Jul 7;11:13967. doi: 10.1038/s41598-021-93443-x (PMC8263699; doi:10.1038/s41598-021-93443-x)
Supplement: Supplementary file 1 — Supplementary Information. [file 41598_2021_93443_MOESM1_ESM.doc]

Supplementary Materials for

**Health economics modeling of antiretroviral interventions amongst HIV serodiscordant couples**

Haisheng Wu ^1, †^, Qiuyan Yu ^2, †^, Liping Ma ^3^, Lin Zhang ^4^, Yuliang Chen ^1^, Pi Guo ^1, #^, Peng Xu ^5, #^

^1^ Department of Preventive Medicine, Shantou University Medical College, No.22 Xinling Road, Shantou 515041, China

^2^ Department of Preventive Medicine, School of Public Health and Management, Wenzhou Medical University, University Town, Wenzhou 325035, China

^3^ Hengrui Pharmaceutical Co., Ltd., No.7 Kunlun Mountain Road, Lianyungang Economic and Technological Development Zone, Jiangsu Province, China

^4^ Zhoukou Center for Disease Control and Prevention, No.10 Taihao Road East Section, Zhoukou City, Henan Province, China

^5^ National Center for STD/AIDS Prevention and Control, Chinese Center for Disease Control and Prevention, No.155 Changbai Road, Beijing 102206, China

^†^ These authors contributed equally in this work and they are the co-first authors.

**^#^ Corresponding author:**

**Pi Guo**, Department of Preventive Medicine, Shantou University Medical College, Shantou 515041, China

E-mail: pguo@stu.edu.cn

**Peng Xu**, National Center for STD/AIDS Prevention and Control, Chinese Center for Disease Control and Prevention, No. 155 Changbai Road, Beijing 102206, China

E-mail: xupeng2007@163.com

**Contents**

**Additional model details**

**Figure S1.** Adjusted yearly transition probabilities stratified by calendar years. The fitted values of yearly adjusted transition probabilities between health states in on-ART period, estimated by MMSM model eliminating the influence of other covariates observed during three time periods (2003-2007, 2007-2011, 2011-2015).

**Figure S2.** Adjusted yearly transition probabilities stratified by age. The fitted values of yearly adjusted transition probabilities between health states in on-ART period, estimated by MMSM model eliminating the influence of other covariates observed during three age groups (<40, 40-50, >50 years).

**Figure S3.** **Outcomes of scenario analysis for rates relationship dissolution.** We explored the sensitivity of model results in a series of scenario regarding the relationship dissolution rate as 10%, 20%, 30%, 40% and 50% respectively. Top panel: ICER and ICUR varied from different rates of relationship dissolution among serodiscordant couples of mid-ART; middle panel: ICER and ICUR varied from different rates of relationship dissolution among serodiscordant couples of early-ART; bottom panel: ICER and ICUR varied from different rates of relationship dissolution among serodiscordant couples of early-ART plus PrEP. The threshold for cost-effectiveness of $3,797-11,391 is shown in the grey band, and the cost-saving threshold was shown by the black dashed line.

**Figure S4.** Outcomes of one-way analysis of mid-ART cohort. We calculated the impacts of specific cost and transmission-related variables on (a) ICER in mid-ART versus late-ART; (b) ICUR in mid-ART versus late-ART. The dashed vertical grey line represents the threshold ($ 3776) for being very cost-effective. These variable change within the range shown in Table 1.

**Figure S5.** Outcomes of one-way analysis about state utility. We calculated the impacts of utility of each health state on (a) ICUR in early-ART versus late-ART; (b) ICUR in mid-ART versus late-ART; (c) ICUR in short-term PrEP + early-ART versus late-ART. The dashed vertical grey line represents the threshold ($ 3776) for being very cost-effective. The utility of each health state changes within the estimated 95% confidence interval as shown in Table 1.

**Figure S6. The relationship between percentage reduction in cost of PrEP drugs and cost-effectiveness.** The threshold for cost-effectiveness of $3,797-11,391 is shown in the grey band.

**Table S1.** Basic characteristics of samples (*n*=2,096) used in the MMSM model.

**Table S2.** CD4 improvement and deterioration during ART for HIV-positive partners. We present hazard ratios (HRs) generated by MMSM model to represent improvement or deterioration in CD4 strata. For improvement in CD4 strata, HRs < 1 therefore indicate delayed time to CD4 improvement; For deterioration in CD4 strata, HRs > 1 indicate delayed time to CD4 improvement.

**Table S3.** Yearly transition probabilities from health states of pre-ART period to states of on-ART period and death. These probabilities were generated from the MMSM model, correcting the effect of factors that had a significant impact on state transitions shown in Table S2.

**Table S4.** Yearly transition probabilities among heath states of on-ART period. These probabilities were generated from the MMSM model, correcting the effect of factors that had a significant impact on state transitions shown in Table S2.

**Table S5.** Yearly transition probabilities among health states of early-ART cohort.

**Table S6.** Yearly transition probabilities among health states of mid-ART cohort.

**Table S7.** Yearly transition probabilities among health states of late-ART cohort.

**Table S8.** Additional base-case inputs parameters of model.

**Table S9.** Estimated yearly direct cost ($) of health states. We referred to the cost paid for both individuals and governments as direct costs, including direct medical cost and direct not medical costs.

**Supplement reference**

**Additional model details**

1. **Disease module**

***1.1 Overview***

The disease module as a part of Markov decision model is a computer-based, state-transition model of HIV treatment and progression in resource-limited settings, with the ability to simultaneously track both HIV-related clinical outcomes and the resources linked with these outcomes [1]. State-transition is referred to the model capturing the history of disease evolution in HIV-positive patients as a series of yearly transitions amongst health status [2]. The clinical course of a HIV-seropositive partner is followed from the time of initial diagnose to 30 years later or death. The disease module recorded the running tally of disease evolution as well as transmission events, state-specific duration and cost, and then deliver the information of transmission events to the transmission module. An HIV-negative partner could enter the disease module after an occurrence of seroconversion. The model-based projections of disease module, including per person life expectancy, quality of life and cost, were calculated form a societal perspective accounted for income loss and time.

***1.2 Health states***

The disease module defined health states to depict HIV-related history, current health status, and resource allocation patterns of HIV-positive partners. These health states are assigned to predict disease evolution (improvement or deterioration of CD4 cell counts), different yearly cost due to state-relevant opportunistic diseases, transmission rates and mortality. Firstly, the disease module divides health states into three periods: treatment-naive (pre-ART), treatment (on-ART), and death. When the patients are in pre-ART period, the opportunistic infection is more likely to occur due to the lack of antiviral drugs. Patients who are offering ART move into on-ART status, where resource consumption levels are higher but both deterioration (CD4 decline) rates of the immune system and mortality rates are lower.

These health states are specifically defined according CD4 cell counts as: S1 (> 500 cells/mm^3^ in pre-ART); S2 (350 to 499 cells/mm^3^ in pre-ART); S3 (200 to 349 cells/mm^3^ in pre-ART); S4 (< 200 cells/mm^3^ in pre-ART); S1' (> 500 cells/mm^3^ on ART); S2' (350 to 499 cells/mm3 on ART); S3' (200 to 349 cells/mm^3^ on ART); S4' (< 200 cells/mm^3^ on ART); and S5 (death).

Upon entering the simulation cohort, HIV patients are randomly assigned to an initial health state according to distributions of patients’ descriptive demographic (age, sex, and CD4 cell count) derived from the real-world database about HIV-serodiscordant couples of Zhoukou city. The disease module allows the flexibility to explore various cohorts based on different intervention strategies, by defining the initial population distribution of age, gender, CD4-based states, clinical features and other characteristics. At the begin of each 1-year cycle, the disease module records the treatment- and CD4-based states of HIV-positive patients, current transmission within marriages and utilizes these attributes to decide the transition probabilities in the subsequent year and seroconversion rates of their partners.

***1.3 Multivariable multi-state Markov model***

In most cases, health resource allocation evaluations are planned for life-long or a long-term period, meaning that problematic health programs or technologies will be made available to clients in need indefinitely. Therefore, disease-oriented health economic evaluations are typically conducted through mathematical models to simulate and record projected outcomes within a predefined health state.

It is important to precisely estimate the rates of disease progression or the transition probabilities between disease states to develop reasonable individual simulation models and mathematical models. Because there is a strong association between CD4 cell counts and the cost of health resources and HIV-caused quality of life [3, 4], a common approach for HIV health economic assessment is to characterize disease progression by a sequence of transitions between CD4-based health states [5, 6]. In this context, a reasonable and reliable health resource allocation need to precisely estimate the CD4 evolution that includes rate of disease progression or the transition probabilities among health states to project the current and future burden for HIV-serodiscordant couples. However, although a variety of recipes for this problem have been utilized to obtain an approximate estimate of HIV disease evolution over time, the univariate manner has always been used to calculate the transition probabilities of health economic evaluations alongside clinical trials, ignoring that HIV patients are a highly heterogeneous population.

We used a multivariable multi-state Markov (MMSM) model, a mathematical model that combines multiple regression models and multiple states Markov model [7, 8], to estimate the yearly transition probabilities among health states of the seropositive. The target of this model was to (1) to explain the heterogeneity of patient characteristics and allow flexible distribution of different clinical stages, (2) estimate the effects of covariates for disease state transition, (3) compare the impacts of each factor on improvement or deterioration of health states, d) obtain a corrected transition matrix that eliminated the impact of some or all of the covariates in the model.

All 2096 eligible individuals from real-world database were used in the MMSM model their basic characteristics were presented in Table S1. The MSMM model would show signs of improvement in disease progression and deterioration in disease progression [8]. We verified some available variables that could affect the disease evolution process. These variables included gender, baseline CD4 (initial CD4 measurement in pre-ART period: <200 cells/mm^3^ vs. >=200 cells/mm^3^), treatment regimen of ART initiation (standard vs. not standard) and age. We also considered impacts caused by the possible temporal period of CD4 measurement (2003-2007, 2007-2011, and 2011-2015). The final MMSM model only contained covariates that have a statistically significant impact on any specified transmissions between CD4-based states.

We assume that the HIV infections can advance or recover from consecutive states while alive, and die from any states [8], indicating that the model allows instantaneous transitions between adjacent health states or between each health state and death. Thus the model of different ART initiation strategies needs to define different instantaneous transitions. Compared with the early-ART, there was no instantaneous transition from S1 (CD4 > 500 cells/mm^3^ at pre-ART) to S1' (CD4 >500 cells/mm^3^ on ART) in the mid-ART, and no instantaneous transition from S2 (CD4 350 to 499 cells/mm^3^ at pre-ART) to S2' (CD4 350 to 499 cells/mm^3^ on ART) and S1 to S1' in the late-ART.

The MMSM model, with the same assumptions as the conventional proportional hazard model, hypothesized that both fixed and time-varying covariates would affect the baseline intensity through a proportional (constant over time) factor [8]. Thus, the MMSM model established with ten instantaneous transitions needed to estimate ten different regression coefficients and ten exponentiated coefficient estimates with a similar interpretation with adjusted hazard ratio in the Cox model, which were estimated by maximum likelihood estimation. For all hypotheses tested, a significance level of *α*= 0.05 was used. Table S2 shows the results of the MMSM model.

Two adjusted transition probability matrices (Tables S3-S4) were finally estimated by the MMSM model. These transition probabilities enclosed in the matrix would be used in the final mathematical model to analyze the cost-effectiveness of different strategies. One of the matrices contains the probability of transferring from CD4-based states before ART treatment to CD4-based states during ART treatment, estimated by CD4 states measured at the initial measurement and CD4 states measured at the first follow-up after ART initiation. Due to the inclusion and exclusion criteria for data collection in the retrospective study, the time interval between CD4 measurements was no more than 12 months. The other matrix contained the transition probabilities between the CD4 states during ART treatment, which were estimated by the follow-up CD4 measurements after ART initiation.

1. **Transmission module**

***2.1 Overview***

The transmission module is a deterministic, non-dynamic model of HIV transmission and its associated clinical and economic impacts [2]. This new module is designed to project the timing and consequences of HIV transmissions within marriages. Given the complexity of the time-dependent transmissions rates as well as efficacy of PrEP drugs there are to model and the database only provided a portion of these partnerships, we also get relevant information for transmission modeling from published literature.

To account for the timing, clinical, life quality, and economic impacts of HIV transmission within marriages, we first simulate the lives of a cohort of index patients. In each year, probabilities of HIV transmission corresponding to each intervention cohort are assigned to each HIV-infected person. These probabilities are used to project the number of transmissions that occur over the risk period of the index case. To calculate the loss of life years, QALYs, productivity and cost gains among the transmitted cases, outcomes of newly-infected patients are compared to outcomes of an equivalent, but uninfected, cohort and discounted to the time in which they occurred. These outcomes are then included in the overall ICER and overall ICUR calculation.

To satisfy the cost-effectiveness framework described above, the transmission module was designed to perform the following three functions: 1) it projects the number and timing of transmission events; 2) it projects the cost gains associated with these transmissions; and 3) it projects the survival losses associated with these transmissions.

***2.2 Outcome projections***

After the number of transmissions each year during the risk period is calculated, the transmission module will need to project the survival and life quality of these individuals, both with and without HIV. To do this, it uses the Markov disease model to determine an average survival trajectory and trajectory of life quality; it then applies this survival trajectory deterministically to the newly-infected cohorts.

***2.3 Adjusted transmissions rates***

The age- and time-depend transmission rates were estimated from real-world databases and published literature. Taking into account the average age of HIV-serodiscordant couples in China, we set the average duration of risk period for the study population to be first 5 years in simulation [2]. Stratified by gender (male-to-female and female-to-male), the transmission rates per year during the risk period was calculated according to equation 1.

$TR=P_{1}\cdot R_{1}+P_{2}\cdot R_{2}$ (1)

Here, *P_1_* and *P_2_* represent the time-dependent proportion of pre-ART group and the proportion of the on-ART group for the specific intervention, respectively. The calculations for *P_1_* and *P_2_* were attributed to the Markov disease model's ability to track the proportion distribution of index patients in each state each year. *R_1_* is the time-varying transmission rates of the treatment-naïve group within HIV-serodiscordant couples, obtained from a nationwide study [9]. *R_2_* represents the transmission rates of treatment group, estimated by real-world database of HIV-serodiscordant couples in Zhoukou city.

1. **Calculating the incremental cost-effectiveness ratio and incremental cost-utility ratio**

Equation 2 shows a general calculation method for the incremental cost-effectiveness ratio (ICER) on a basis of the average per person cumulative health resource costs and the life expectancy under two different strategies. Here, *C_0_* is the average per person health resource costs of the cohort accrued under baseline strategy, *Y_0_* is the per person life expectancy (in life-years) of the cohort under baseline strategy, and *C_1_* and *Y_1_* are the respective results under a concerned strategy (e.g. early-ART). Analogously, the incremental cost-utility ratio (ICUR) of an intervention strategy is calculated based on the average per person accumulated healthcare costs and the quality-adjusted life years (QALYs) under two different strategies, as shown in Equation 3 below, and *Q_1_* and *Q_0_* are the respective QALYs.

$ICER=\frac{C_{1}-C_{0}}{Y_{1}-Y_{0}}$ (2)

$ICUR=\frac{C_{1}-C_{0}}{Q_{1}-Q_{0}}$ (3)

To additionally include the impact of productivity loss and transmissions within marriages, we amend this formula:

$ICER=\frac{(C_{1}+CP_{1}+CT_{1}+CTP_{1})-(C_{0}+CP_{0}+CT_{0}+CTP_{0})}{(Y_{1}-YT_{1})-(Y_{0}-YT_{0})}$ (4)

$ICUR=\frac{(C_{1}+CP_{1}+CT_{1}+CTP_{1})-(C_{0}+CP_{0}+CT_{0}+CTP_{0})}{(Q_{1}-QT_{1})-(Q_{0}-QT_{0})}$ (5)

The parameters of the Equation 4-5 above can be calculated for any strategy:

***CP*:** productivity loss in index partners. Local per capita income, general unemployment rates, and HIV population unemployment rates were used to calculate the loss of productivity due to HIV for specific strategies.

***CT*:** cost due to seroconversion events among the original negative partners. We first projected the number of transmissions under that strategy and when they occurred (for purposes of discounting), then subtracted the costs the newly-infected individuals would accrue without the disease from the costs they will accrue with the disease.

***CTP*:** productivity loss due to transmissions, similarly to the *CP*.

***YT*:** loss of life expectancy due to transmissions, is calculated by comparing the survival of these individuals to whom HIV was transmitted and when they occurred with and without the disease.

***QT*:** loss of QALYs due to transmissions, is calculated by comparing the QALYs of these individuals to whom HIV was transmitted and when they occurred (for discounting purposes) with and without the disease.

**Figure S1. Adjusted yearly transition probabilities stratified by calendar years.** The fitted values of yearly adjusted transition probabilities between health states in on-ART period, estimated by MMSM model eliminating the influence of other covariates observed during three time periods (2003-2007, 2007-2011, and 2011-2015).


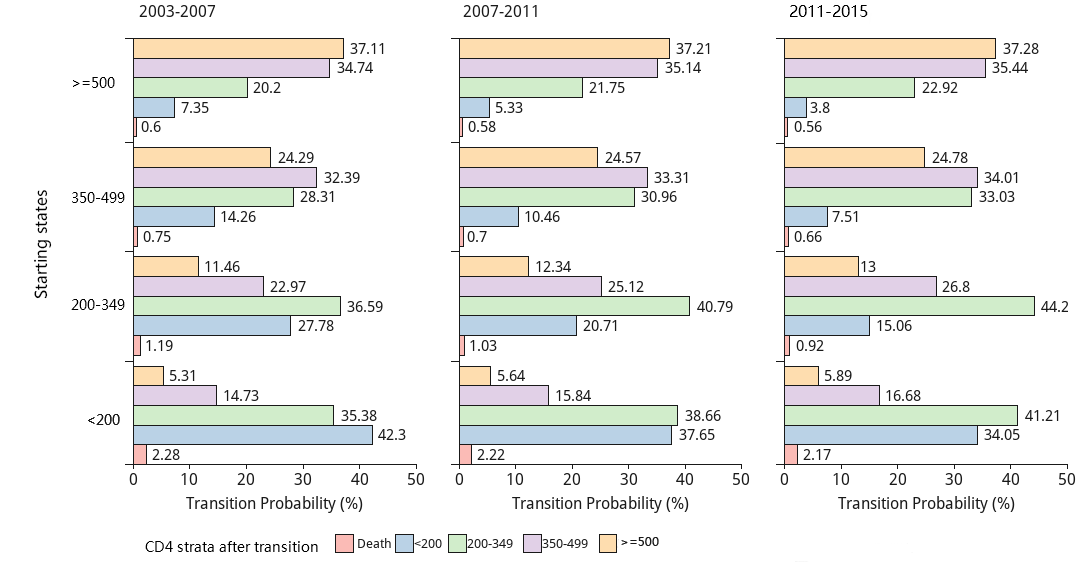


**Figure S2. Adjusted yearly transition probabilities stratified by age.** The fitted values of yearly adjusted transition probabilities between health states in on-ART period, estimated by MMSM model eliminating the influence of other covariates observed during three age groups (<40, 40-50, and >50 years).


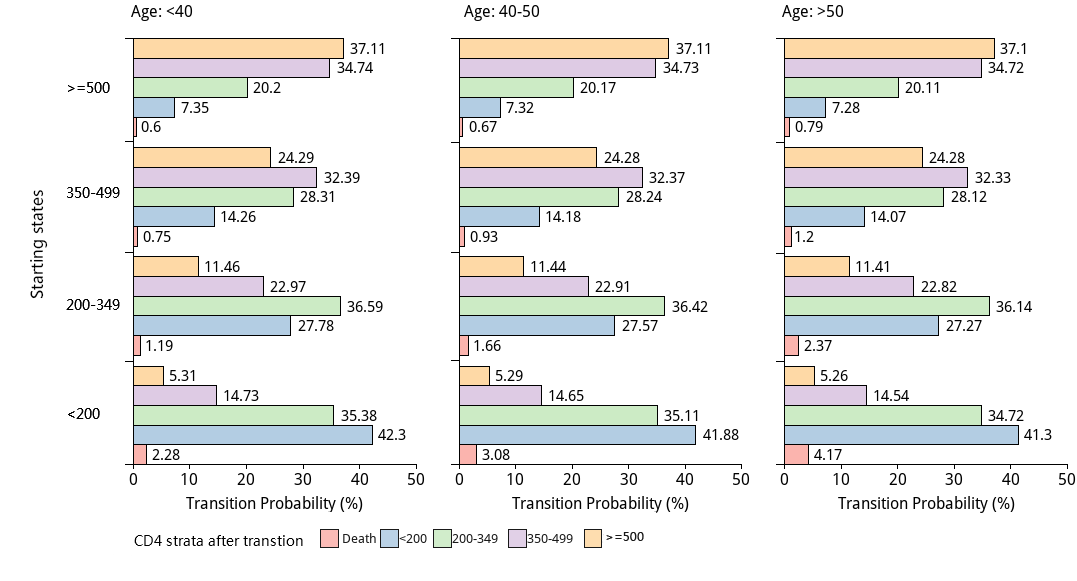


**Figure S3. Outcomes of scenario analysis for rates relationship dissolution.** We explored the sensitivity of model results in a series of scenario regarding the relationship dissolution rate as 10%, 20%, 30%, 40% and 50% respectively. Top panel: ICER and ICUR varied from different rates of relationship dissolution among serodiscordant couples of mid-ART; middle panel: ICER and ICUR varied from different rates of relationship dissolution among serodiscordant couples of early-ART; bottom panel: ICER and ICUR varied from different rates of relationship dissolution among serodiscordant couples of early-ART plus PrEP. The threshold for cost-effectiveness of $3,797-11,391 is shown in the grey band, and the cost-saving threshold was shown by the black dashed line.





**Figure S4. Outcomes of one-way analysis of mid-ART cohort.** We calculated the impacts of specific cost and transmission-related variables on (a) ICER in mid-ART versus late-ART; (b) ICUR in mid-ART versus late-ART. The dashed vertical grey line represents the threshold ($ 3776) for being very cost-effective. These variable change within the range shown in Table 1.


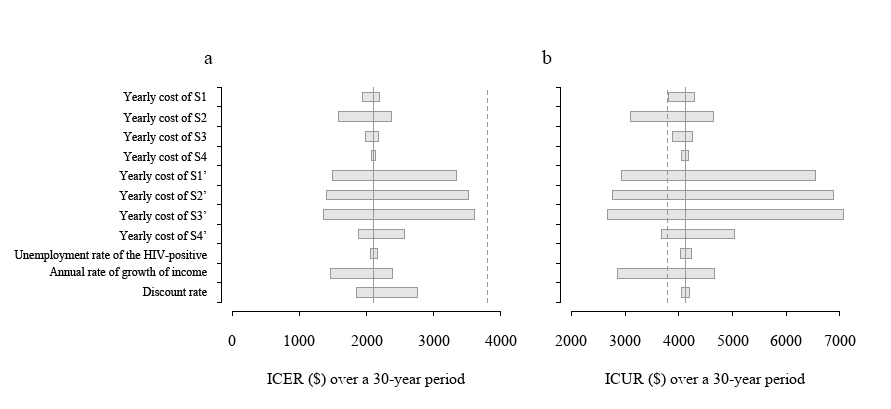


**Figure S5. Outcomes of one-way analysis about state utility.** We calculated the impacts of utility of each health state on (a) ICUR in early-ART versus late-ART; (b) ICUR in mid-ART versus late-ART; (c) ICUR in short-term PrEP + early-ART versus late-ART. The dashed vertical grey line represents the threshold ($ 3776) for being very cost-effective. The utility of each health state changes within the estimated 95% confidence interval as shown in Table 1.


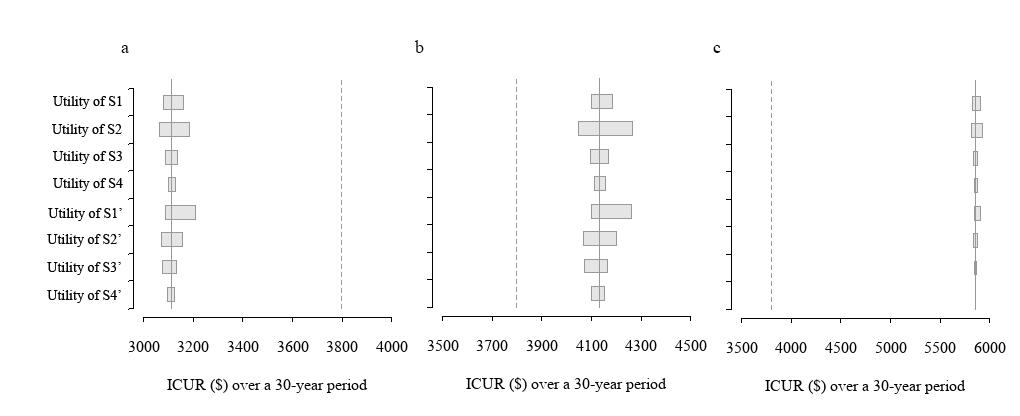


**Figure S6. The relationship between percentage reduction in cost of PrEP drugs and ICER/ICUR.** The threshold for cost-effectiveness of $3,797-11,391 is shown in the grey band.


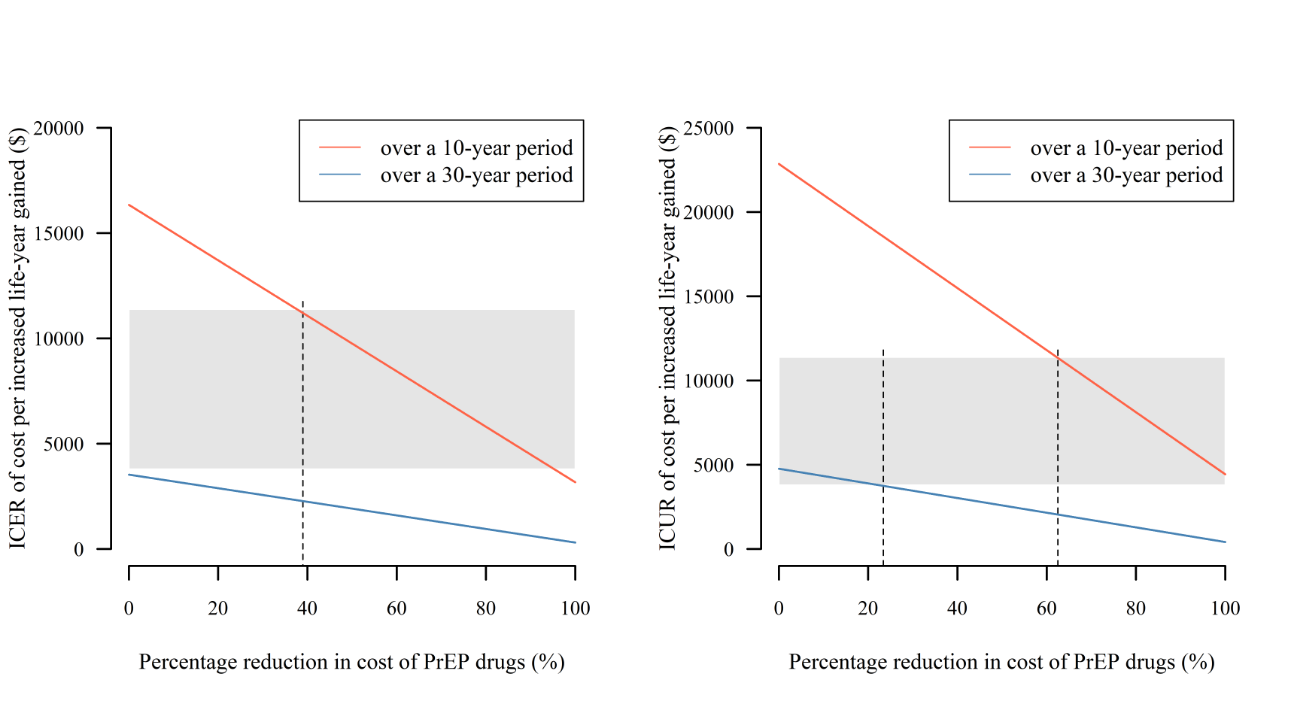


**Table S1. Basic characteristics of samples (*n*=2,096) used in the MMSM model.**

| Characteristics | Number |
| --- | --- |
|
| Males, number (%) | 1201 (54.69) |
| Median age, years (IQR) | 43 (38-50) |
| Baseline CD4 (cell count/mm ^3^), number (%) |  |
| *>=500* | 228 (10.38) |
| *350-499* | 310 (14.12) |
| *200-349* | 694 (31.60) |
| *<200* | 228 (43.90) |
| Year of first ART, number (%) |  |
| *2003-2007* | 907 (41.30) |
| *2007-2011*  *2011-2015* | 613 (27.92)  676 (30.78) |
| Baseline therapy, number (%) |  |
| *Standard* | 1234 (56.19) |
| *Not standard* | 962 (43.81) |

**Table S2. CD4 improvement and deterioration during ART for HIV-positive partners. We present hazard ratios (HRs) generated by MMSM model to represent improvement or deterioration in CD4 strata. For improvement in CD4 strata, HRs < 1 therefore indicate delayed time to CD4 improvement; For deterioration in CD4 strata, HRs > 1 indicate delayed time to CD4 improvement.**

|  | Male  (*vs.* female) | CD4 (>200)  (*vs.* <200) | Calendar year | Age | Standard therapy  (*vs.* not standard) |
| --- | --- | --- | --- | --- | --- |
| Transitions |
| Improvement of CD4 strata | |  |  |  |  |
| *S4' to S3'* | 0.85(0.75,0.96) | 1.39(1.23,1.57) | -- | -- | 1.50(1.33,1.68) |
| *S3' to S2'* | -- | 1.38(1.25,1.54) | -- | -- | 1.20(1.08,1.33) |
| *S2' to S1'* | -- | -- | -- | -- | 1.33(1.19,1.49) |
|  |  |  |  |  |  |
| Deterioration of CD4 strata | |  |  |  |  |
| *S3' to S4'* | -- | -- | 0.70(0.62,0.79) | -- | -- |
| *S2' to S3'* | 1.23(1.09,1.38) | 0.85(0.76,0.96) | 0.73(0.66,0.82) | -- | -- |
| *S1' to S2'* | -- | 0.61(0.54,0.70) | 0.78(0.70,0.88) | -- | 1.16(1.03,1.31) |
| *S4' to death* | -- | -- | 0.62(0.42,0.93) | 1.65(1.17,2.32) | -- |
| *S3' to death* | -- | -- | -- | 2.28(1.06,4.92) | -- |
| *S2' to death* | -- | -- | -- | -- | -- |
| *S1' to death* | -- | -- | -- | -- | -- |

‘--’ means the HRs is no significant difference with 1.

**Table S3. Yearly transition probabilities from health states of pre-ART period to states of on-ART period and death.** These probabilities were generated from the MMSM model, correcting the effect of factors that had a significant impact on state transitions shown in Table S2.

| States of Pre-ART period | States of on-ART period | | | | |
| --- | --- | --- | --- | --- | --- |
|  | S4' | S3' | S2' | S1' | Death |
| S4 | 0.379 | 0.260 | 0.127 | 0.039 | 0.240 |
| S3 | 0.173 | 0.350 | 0.240 | 0.123 | 0.150 |
| S2 | 0.127 | 0.349 | 0.269 | 0.216 | 0.050 |
| S1 | 0.037 | 0.169 | 0.207 | 0.548 | 0.050 |
| Death | 0.000 | 0.000 | 0.000 | 0.000 | 1.000 |

**Table S4. Yearly transition probabilities among heath states of on-ART period. These probabilities were generated from the MMSM model, correcting the effect of factors that had a significant impact on state transitions shown in Table S2.**

| Pre-transition states | Post-transition states | | | | |
| --- | --- | --- | --- | --- | --- |
|  | S4' | S3' | S2' | S1' | Death |
| S4 | 0.343 | 0.369 | 0.188 | 0.076 | 0.024 |
| S3 | 0.157 | 0.380 | 0.285 | 0.166 | 0.012 |
| S2 | 0.082 | 0.294 | 0.327 | 0.290 | 0.007 |
| S1 | 0.036 | 0.186 | 0.314 | 0.459 | 0.006 |
| Death | 0.000 | 0.000 | 0.000 | 0.000 | 1.000 |

**Table S5. Yearly transition probabilities among health states of early-ART cohort.**

| Pre-transition states | Post-transition states | | | | | | | | |
| --- | --- | --- | --- | --- | --- | --- | --- | --- | --- |
|  | S4 | S3 | S2 | S1 | S4' | S3' | S2' | S1' | Death |
| S4 | 0.000 | 0.000 | 0.000 | 0.000 | 0.336 | 0.258 | 0.127 | 0.039 | 0.240 |
| S3 | 0.000 | 0.000 | 0.000 | 0.000 | 0.173 | 0.314 | 0.240 | 0.123 | 0.150 |
| S2 | 0.000 | 0.000 | 0.000 | 0.000 | 0.127 | 0.349 | 0.258 | 0.216 | 0.050 |
| S1 | 0.000 | 0.000 | 0.000 | 0.000 | 0.037 | 0.169 | 0.207 | 0.537 | 0.050 |
| S4' | 0.000 | 0.000 | 0.000 | 0.000 | 0.364 | 0.356 | 0.178 | 0.072 | 0.030 |
| S3' | 0.000 | 0.000 | 0.000 | 0.000 | 0.161 | 0.377 | 0.283 | 0.166 | 0.014 |
| S2' | 0.000 | 0.000 | 0.000 | 0.000 | 0.083 | 0.292 | 0.327 | 0.291 | 0.008 |
| S1' | 0.000 | 0.000 | 0.000 | 0.000 | 0.036 | 0.184 | 0.313 | 0.461 | 0.006 |
| Death | 0.000 | 0.000 | 0.000 | 0.000 | 0.000 | 0.000 | 0.000 | 0.000 | 1.000 |

**Table S6. Yearly transition probabilities among health states of mid-ART cohort.**

| Pre-transition states | Post-transition states | | | | | | | | |
| --- | --- | --- | --- | --- | --- | --- | --- | --- | --- |
|  | S4 | S3 | S2 | S1 | S4' | S3' | S2' | S1' | Death |
| S4 | 0.000 | 0.000 | 0.000 | 0.000 | 0.336 | 0.258 | 0.127 | 0.039 | 0.240 |
| S3 | 0.000 | 0.000 | 0.000 | 0.000 | 0.173 | 0.314 | 0.240 | 0.123 | 0.150 |
| S2 | 0.000 | 0.000 | 0.000 | 0.000 | 0.127 | 0.349 | 0.258 | 0.216 | 0.050 |
| S1 | 0.050 | 0.100 | 0.200 | 0.600 | 0.000 | 0.000 | 0.000 | 0.000 | 0.050 |
| S4' | 0.000 | 0.000 | 0.000 | 0.000 | 0.364 | 0.356 | 0.178 | 0.072 | 0.030 |
| S3' | 0.000 | 0.000 | 0.000 | 0.000 | 0.161 | 0.377 | 0.283 | 0.166 | 0.014 |
| S2' | 0.000 | 0.000 | 0.000 | 0.000 | 0.083 | 0.292 | 0.327 | 0.291 | 0.008 |
| S1' | 0.000 | 0.000 | 0.000 | 0.000 | 0.036 | 0.184 | 0.313 | 0.461 | 0.006 |
| Death | 0.000 | 0.000 | 0.000 | 0.000 | 0.000 | 0.000 | 0.000 | 0.000 | 1.000 |

**Table S7. Yearly transition probabilities among health states of late-ART cohort.**

| Pre-transition states | Post-transition states | | | | | | | | |
| --- | --- | --- | --- | --- | --- | --- | --- | --- | --- |
|  | S4 | S3 | S2 | S1 | S4' | S3' | S2' | S1' | Death |
| S4 | 0.000 | 0.000 | 0.000 | 0.000 | 0.336 | 0.258 | 0.127 | 0.039 | 0.240 |
| S3 | 0.000 | 0.000 | 0.000 | 0.000 | 0.173 | 0.314 | 0.240 | 0.123 | 0.150 |
| S2 | 0.050 | 0.120 | 0.660 | 0.120 | 0.000 | 0.000 | 0.000 | 0.000 | 0.050 |
| S1 | 0.050 | 0.100 | 0.200 | 0.600 | 0.000 | 0.000 | 0.000 | 0.000 | 0.050 |
| S4' | 0.000 | 0.000 | 0.000 | 0.000 | 0.364 | 0.356 | 0.178 | 0.072 | 0.030 |
| S3' | 0.000 | 0.000 | 0.000 | 0.000 | 0.161 | 0.377 | 0.283 | 0.166 | 0.014 |
| S2' | 0.000 | 0.000 | 0.000 | 0.000 | 0.083 | 0.292 | 0.327 | 0.291 | 0.008 |
| S1' | 0.000 | 0.000 | 0.000 | 0.000 | 0.036 | 0.184 | 0.313 | 0.461 | 0.006 |
| Death | 0.000 | 0.000 | 0.000 | 0.000 | 0.000 | 0.000 | 0.000 | 0.000 | 1.000 |

**Table S8. Additional base-case inputs parameters of model.**

| Variable | Base-case value for male | Base-case value  for male | Range |
| --- | --- | --- | --- |
|  |  |
| Transmission rates of early-ART (no./100 person-year) |  |  |  |
| *The 1st year* | 2.30 | 2.30 | Base case × 0.5-2.0 |
| *The 2nd year* | 0.25 | 0.29 | Base case × 0.5-2.0 |
| *The 3rd year* | 0.25 | 0.29 | Base case × 0.5-2.0 |
| *The 4th year* | 0.25 | 0.29 | Base case × 0.5-2.0 |
| *The 5th year* | 0.29 | 0.29 | Base case × 0.5-2.0 |
| Transmission rates of mid-ART (no./100 person-year) |  |  |  |
| *The 1st year* | 2.51 | 2.49 | Base case × 0.5-2.0 |
| *The 2nd year* | 0.44 | 0.46 | Base case × 0.5-2.0 |
| *The 3rd year* | 0.37 | 0.40 | Base case × 0.5-2.0 |
| *The 4th year* | 0.35 | 0.38 | Base case × 0.5-2.0 |
| *The 5th year* | 0.32 | 0.35 | Base case × 0.5-2.0 |
| Transmission rates of late-ART (no./100 person-year) |  |  |  |
| *The 1st year* | 2.78 | 2.76 | Base case × 0.5-2.0 |
| *The 2nd year* | 0.69 | 0.70 | Base case × 0.5-2.0 |
| *The 3rd year* | 0.54 | 0.55 | Base case × 0.5-2.0 |
| *The 4th year* | 0.49 | 0.51 | Base case × 0.5-2.0 |
| *The 5th year* | 0.41 | 0.43 | Base case × 0.5-2.0 |

**Table S9.** **Estimated yearly direct cost ($) of health states.** We referred to the cost paid for both individuals and governments as direct costs, including direct medical cost and direct not medical costs.

| Category | | CD4<200 | 200<CD4<350 | 350<CD4<500 | CD4>500 |
| --- | --- | --- | --- | --- | --- |
| Direct medical cost ($) | |  |  |  |  |
| *Pre-testing* | | 12.4 | 10.7 | 10.2 | 9.4 |
| *CD4 testing* | | 53.6 | 51.8 | 48.8 | 45.6 |
| *VL testing* | | 97.9 | 94.1 | 86.8 | 77.9 |
| *First-line drugs* | | 330.7 | 347.4 | 356.5 | 359.8 |
| *Second-line drugs* | | 362.0 | 312.8 | 285.9 | 276.3 |
| *Follow-up* | | 26.8 | 26.2 | 26.0 | 25.7 |
| *Outpatients* | | 1041.9 | 1587.6 | 1205.9 | 963.9 |
| *Hospitalization* | | 388.2 | 457.0 | 471.5 | 391.0 |
| Direct not medical costs ($) | |  |  |  |  |
| *Transportation* | 97.6 | | 116.0 | 136.8 | 155.5 |
| *Caregiver* | 226.6 | | 205.1 | 44.8 | 61.3 |
| *Nutrition* | 121.7 | | 110.9 | 81.1 | 74.2 |
| *Working hours loss* | 569.7 | | 476.2 | 603.5 | 628.4 |
| *Supervision* | 51.5 | | 43.7 | 43.1 | 42.4 |
| *Compensation for follow-up* | 24.4 | | 20.7 | 20.3 | 19.8 |
| *Staff fee for ART drugs administration* | 21.5 | | 18.2 | 18.0 | 17.7 |
| Total ($) |  | |  |  |  |
| *On-ART* | 3426.4 | | 3878.5 | 3439.1 | 3148.9 |

**Supplement references**

1. Jacobsen MM, Walensky RP. Modeling and Cost-Effectiveness in HIV Prevention. Curr HIV/AIDS Rep. 2016 Feb;13(1):64-75. doi: 10.1007/s11904-016-0303-2.
2. Walensky RP, Ross EL, Kumarasamy N, et al. Cost-Effectiveness of HIV Treatment as Prevention in Serodiscordant Couples. N Engl J Med. 2013 Oct 31;369(18):1715-25. doi: 10.1056/NEJMsa1214720.
3. Kauf TL, Roskell N, Shearer A, et al. A predictive model of health state utilities for HIV patients in the modern era of highly active antiretroviral therapy. Value Health. 2008 Dec;11(7):1144-53. doi: 10.1111/j.1524-4733.2008.00326.x.
4. Anis AH, Nosyk B, Sun H, et al; for the OPTIMA Team. Quality of life of patients with advanced HIV/AIDS: measuring the impact of both AIDS-defining events and non-AIDS serious adverse events. J Acquir Immune Defic Syndr. 2009 Aug 15;51(5):631-9. doi: 10.1097/QAI.0b013e3181a4f00d.
5. Walensky RP, Freedberg KA, Weinstein MC, Paltiel AD. Cost-effectiveness of HIV testing and treatment in the United States. Clin Infect Dis. 2007 Dec 15;45 Suppl 4:S248-54. doi: 10.1086/522546.
6. Long EF, Brandeau ML, Owens DK. The cost-effectiveness and population outcomes of expanded HIV screening and antiretroviral treatment in the United States. Ann Intern Med. 2010 Dec 21;153(12):778-89. doi: 10.7326/0003-4819-153-12-201012210-00004.
7. Mitchell K M, Lépine, Aurélia, Terris-Prestholt F, et al. Modelling the impact and cost-effectiveness of combination prevention amongst HIV serodiscordant couples in Nigeria. AIDS. 2015 Sep 24;29(15):2035-44. doi: 10.1097/QAD.0000000000000798.
8. Nosyk B , Min J , Lima V D , et al. HIV-1 disease progression during highly active antiretroviral therapy: an application using population-level data in British Columbia: 1996-2011.[J]. Journal of Acquired Immune Deficiency Syndromes, 2013, 63(5):653. doi: 10.1097/QAI.0b013e3182976891
9. Jia Z, Mao Y, Zhang F. Antiretroviral therapy to prevent HIV transmission in serodiscordant couples in China (2003-11): a national observational cohort study. Lancet. 2013 Oct 5;382(9899):1195-203. doi: 10.1016/S0140-6736(12)61898-4.
